# Distribution of injected fat-soluble vitamins in plasma and tissues of nursery pigs

**DOI:** 10.5713/ajas.19.0987

**Published:** 2020-04-12

**Authors:** Young Dal Jang, Mikayla J. Rotering, Paige K. Isensee, Kirsten A. Rinholen, Carli J. Boston-Denton, Paige G. Kelley, Robert L. Stuart

**Affiliations:** 1 Department of Animal and Food Science, University of Wisconsin-River Falls, River Falls, WI 54022, USA; 2 Stuart Products Inc., Bedford, TX 76022-6297, USA

**Keywords:** Fat-soluble Vitamins, Injection, Nursery Pigs, Plasma, Tissue Concentrations

## Abstract

**Objective:**

The objective of this experiment was to investigate the effects of fat-soluble vitamin injection on plasma and tissue vitamin status in nursery pigs.

**Methods:**

A total of 16 pigs (initial body weight: 7.15±1.1 kg) were allotted to 2 treatments at d 7 post-weaning. Pigs were fed a corn-soybean meal-based basal diet with no supplemental vitamin A and i.m. injected with 300,000 IU of retinyl palmitate, 900 IU of d-α-tocopherol and 30,000 IU of vitamin D_3_ with control pigs having no vitamin injection. Blood (d 0, 3, 7, and 14 post-injection) and tissue samples (liver, brain, heart, lung, and muscle; d 7 and 14 post-injection) were collected from pigs. Retinyl palmitate, retinol, and α-tocopherol concentrations were analyzed in plasma and tissues, while plasma was assayed for 25-hydroxycholecalciferol (25-OHD_3_).

**Results:**

Plasma retinol and 25-OHD_3_ concentrations increased by the vitamin injection from d 3 to 14 post-injection (p<0.05) whereas plasma retinyl palmitate was detected only in the vitamin treatment at d 3 and 7 post-injection (115.51 and 4.97 μg/mL, respectively). Liver retinol, retinyl palmitate, and retinol+retinyl palmitate concentrations increased by retinyl palmitate injection at d 7 and 14 post-injection (p<0.05) whereas those were not detected in the other tissues. The d-α-tocopherol injection increased α-tocopherol concentrations in plasma at d 3 and 7 post-injection (p<0.05) and in liver, heart (p<0.10), and muscle (p<0.05) at d 7 post-injection.

**Conclusion:**

Fat-soluble vitamin injection increased plasma status of α-tocopherol, retinol, retinyl palmitate and 25-OHD_3_. As plasma levels decreased post-injection, vitamin A level in liver and vitamin E level in muscle, heart and liver increased. The α-tocopherol found in plasma after injection was distributed to various tissues but retinyl palmitate only to the liver.

## INTRODUCTION

Fat-soluble vitamins are important micronutrients in pigs for normal physiological functions related to vision, immunity, bone integrity, and antioxidant properties [1]. Piglets are born with purportedly low levels of fat-soluble vitamins in plasma [2,3] and plasma levels are reported to either increase or be relatively constant through weaning [4,5]. It has been demonstrated that fat-soluble vitamin injection with retinyl palmitate, vitamin D_3_, and d-α-tocopherol increased plasma retinyl palmitate, 25-hydroxycholecalciferol (25-OHD_3_) and α-tocopherol concentrations of newborn pigs [4,5], and those were declined from the peaks of plasma vitamin levels after injection with different rates of disappearance between vitamin A, D_3_, and E. Jang et al [5,6] reported that intramuscular injection of fat-soluble vitamins to newborn piglets increased plasma vitamin levels greater than oral administration and that the response of pigs to vitamin A administration was obviously less than that to vitamin D_3_ and E administration. These results may be attributed to the fact that liver is a main organ that regulates vitamin A storage and release into the blood [7]. Therefore, it is important to understand metabolism and distribution of fat-soluble vitamins in blood and body tissues after administration. However, these previous studies showed only plasma vitamin status of pigs and there are limited data available that investigate fat-soluble vitamin metabolite profiles in various tissues of pigs as well as in blood.

Therefore, the objective of this study was to evaluate the effects of fat-soluble vitamin injection on fat-soluble vitamin distribution in plasma and critically important tissues (liver, brain, lung, heart, and muscle) of nursery pigs.

## MATERIALS AND METHODS

### Animal care

All procedures used in this study were approved by the Institutional Animal Care and Use Committee of University of Wisconsin-River Falls (Protocol #17-18-8). The experiment was conducted in the nursery facility at Mann Valley Farm of University of Wisconsin-River Falls (WI, USA).

### Animals, experimental design, and treatments

A total of 32 pigs, weaned at 19.4±0.5 d of age, were fed a common diet (corn-soybean meal-based) without vitamin A for 7 d after weaning to ensure their health and normal growth. At d 7 post-weaning, a subset of 16 pigs were selected from the larger group of pigs based upon uniformity of body weight as well as their general appearance and wellbeing. The pigs (initial body weight: 7.15±1.1 kg; Yorkshire× Duroc, Yorkshire×Yorkshire; 8 barrows and 8 gilts) were allotted to 2 treatments (n = 8 per treatment; 4 barrows and 4 gilts) in 4 pens based on body weight, breed, litter, and sex. The pigs were housed with 4 pigs per pen in which there were 2 pigs from each treatment with a same sex. Treatments were i) Control, No fat-soluble vitamin injection; and ii) VITA, i.m. injection with 3 mL of 100,000 IU retinyl-palmitate, 300 IU d-α-tocopherol and 10,000 IU vitamin D_3_ per mL (Vital E-A+D; Stuart Products, Inc., Bedford, TX USA).

### Diets, housing, and vitamin administration

All pigs were fed a common corn-soybean meal based basal diet without vitamin A *ad libitum* with free access to water throughout the entire experimental period for 14 d after injection. Vitamin A was not added in the vitamin premix when the common experimental diet was mixed to exclude a potential effect of vitamin A from the diet that may affect the vitamin A level in the liver. All other essential nutrients were in slight excess of the NRC [8] requirement estimates (Table 1). The pigs were housed in raised-deck nursery pens (1.32×1.63 m^2^) with plastic flooring in an environmentally controlled nursery facility and weighed at d 7 and 14 post-injection. At the beginning of the experiment, each pig in the VITA treatment was injected with fat-soluble vitamins in the trapezius muscle.

### Data and sample collection

Prior to initiation of experiment (d 0), four pigs were euthanized and tissues were collected for determination of initial fat-soluble vitamin levels in tissues. Blood samples were collected from the jugular vein into a glass tube containing K3 ethylenediaminetetraacetic acid at d 0 (initial; before vitamin administration), 3, 7, and 14 post-injection for determination of plasma fat-soluble vitamin levels. Blood samples were centrifuged at 2,200 g for 20 minutes at 4°C; plasma samples were then aliquoted into microtubes and stored at −80°C until analysis. At d 7 post-injection, three pigs (2 barrows and 1 gilt) per treatment were selected based on the body weight after bleeding for tissue necropsy, and liver, brain, heart, lung, and muscle (ham) samples were collected. At d 14 post-injection, the other 3 pigs (2 barrows and 1 gilt) were sacrificed for tissue sample collection. The tissue samples were flash-frozen in liquid N and stored at −80°C with plasma samples until further analysis. Plasma and tissue samples were sent to Iowa State University Veterinary Diagnostic Laboratory and analyzed for 25-OHD_3_ (plasma only), α-tocopherol, retinyl palmitate and retinol concentrations. Retinol+ retinyl palmitate concentrations in liver were calculated by summing retinol and retinyl palmitate concentrations in liver after conversion to retinol equivalents.

### Statistical analysis

Growth data (body weight and average daily gain) were analyzed by analysis of variance (ANOVA) for a randomized complete block design using PROC MIXED of SAS (version 9.2; SAS Inst. Inc., Cary, NC, USA). Models included the treatment as a fixed effect and the replicate within a pen, pen, and pen×treatment as random effects. All plasma data were subjected to repeated measures ANOVA to detect the effect of treatment, day, and day×treatment interaction using PROC MIXED of SAS with a heterogeneous autoregressive covariance structure and the replicate within a pen, pen and pen×treatment as random effects. All tissue data at d 7 and 14 post-injection were analyzed by ANOVA using PROC MIXED of SAS. Models included the treatment as a fixed effect and the replicate as a random effect. The experimental unit was the individual pig. Assay values below the limit of analytical detection were treated as missing values. Statistical outliers within each treatment and day were identified using the Grubb’s test outlier calculator (GraphPad Software, San Diego, CA, USA) and excluded from the data analysis. Only one brain α-tocopherol concentration sample was removed from the control treatment as an outlier. Due to a significant difference between treatments in the initial plasma retinol concentrations, the d 0 (initial) value was used as a covariate. Least squares means were separated using the PDIFF option of SAS. Statistical differences were considered significant at p<0.05 and tendency at p<0.10.

## RESULTS

### Growth performance

Because all pigs with 2 treatments were housed in a pen, average daily feed intake per pen was calculated. The average daily feed intake for d 0 to 7 and d 7 to 14 post-injection were 0.366 and 0.781 kg/pig/d, respectively. There were no differences in body weight and average daily gain between treatments throughout the overall experimental period (Table 2).

### Plasma vitamin concentrations

The VITA treatment had greater plasma retinol concentrations than the control treatment from d 3 to 14 post-injection (p<0.05; Table 3). Retinyl palmitate was detected in the VITA treatment only at d 3 and 7 post-injection, and the concentrations at d 3 post-injection were 23 times greater than that of d 7 post-injection. The VITA treatment had greater plasma concentrations of α-tocopherol at d 3 and 7 post-injection (p<0.05) and 25-OHD_3_ from d 3 to 14 post-injection (p<0.05) than the control treatment.

### Liver vitamin A concentrations

Retinyl palmitate injection increased liver concentrations of retinol, retinyl palmitate and retinol+retinyl palmitate at d 7 and 14 post-injection over the control treatment (p<0.05; Table 4). Retinol and retinyl palmitate were not detected in any of the other tissues.

### Tissue α-tocopherol concentrations

The d-α-tocopherol injection increased α-tocopherol concentrations in liver (p = 0.07; tendency) and muscle (p<0.05) over the control treatment at d 7 post-injection (Table 5). The d-α-tocopherol injection tended to increase α-tocopherol concentrations in heart (p = 0.09; tendency) at d 7 and 14 post-injection. There were no differences in brain and lung α-tocopherol concentrations between the treatments at d 7 and 14 post-injection. The α-tocopherol concentrations were reduced from d 7 to 14 post-injection except for brain showing no change.

## DISCUSSION

This study focused primarily on fat-soluble vitamin A and E distribution in blood and tissues of nursery pigs. The initial plasma and tissue vitamin values were considered as baseline values as all pigs were fed a common nursery diet without vitamin A. Growth performance of pigs was measured (body weight and growth rate) and there was no difference between the pigs administered with fat-soluble vitamins by i.m. injection and the control pigs. This result indicated that an i.m. injection of fat-soluble vitamins did not cause any negative impact on piglet growth, which agreed with previous studies [4–6].

When retinyl palmitate was administered to pigs by i.m. injection, plasma retinol concentrations increased. This result indicated that high amount of vitamin A (retinyl palmitate) injection had a potential to enhance plasma vitamin A status. However, Jang et al [5] reported that 40,000 IU of retinyl palmitate injection to newborn piglets did not change plasma retinol concentrations even though plasma retinyl palmitate concentrations in the injection treatment were greater than the non-injection treatment until d 4 post-injection. In the current study, 300,000 IU of retinyl palmitate was injected to nursery pigs and the result indicated that plasma retinol concentrations could increase when high amount of retinyl palmitate was administered to pigs. At d 3 and 7 post-injection, retinyl palmitate was detected in plasma and then became non-detectable at d 14 post-injection. Once vitamin A ester is injected, the retinyl ester with short chain acyl group (acetate, propionate, etc.) needs to be converted to retinyl esters with long-chain acyl group (palmitic acid, stearic acid, etc.), and then stored in the liver in the form of retinyl esters, primarily palmitate [9]. Majchrzak et al [10] reported that retinol and retinyl palmitate accounted for approximately 70% of total vitamin A in the liver of pigs. Retinol (vitamin A alcohol) is released from the liver into the bloodstream and travels via the bloodstream when it is needed [1]. Therefore, this result indicated that retinyl palmitate was still circulating in blood until d 7 post-injection, disappeared from the blood before d 14 post-injection resulting in non-detectable in the plasma, and then found in the liver as greater concentrations of retinol, retinyl palmitate and retinol+retinyl palmitate in the liver were observed in the VITA treatment than the control at d 7 and 14 post-injection in the current study.

Interestingly, retinol and retinyl palmitate were not detected in any other tissues except for liver. Sun et al [11] reported tissue retinyl ester concentrations (liver, lung, kidney, adrenal gland, and spleen) in weaning pigs administered orally with 0 to 30 mg of vitamin A in the form of retinyl acetate in which liver retinyl ester concentrations were detected from 5.1 to 25.6 μg/g whereas lung had only 0.003 to 0.005 μg/g. These lung values were below the detection limit of the vitamin A analysis in the current study. This result indicates that liver is a primary organ of vitamin A storage.

Plasma 25-OHD_3_ and α-tocopherol concentrations increased by vitamin D_3_ and d-α-tocopherol injection, which agreed with the results demonstrated in suckling and nursery pigs by Jang et al [4–6]. Plasma vitamin D and E levels decreased from d 3 to 14 post-injection as those were metabolized. However, the disappearance rate was different between vitamin D and E. In plasma 25-OHD_3_ concentrations, the greater values in the VITA treatment than the control were still maintained until d 14 post-injection whereas plasma α-tocopherol concentrations were greater in the VITA treatment than the control only until d 7 post-injection. These results indicated that α-tocopherol had a more rapid disappearance rate than 25-OHD_3_, which agreed with Jang et al [5]. A corresponding increase in tissue α-tocopherol may explain the rapid decline in plasma concentrations.

Regarding to tissue α-tocopherol concentrations, d-α-tocopherol injection increased α-tocopherol concentrations in liver, heart and muscle except for brain and lung. This result agreed with Pardo [12] that d-α-tocopherol injection to weaning pigs increased tissue α-tocopherol concentrations in liver, heart, muscle, and lung (no brain data). Interestingly, there was no difference in brain α-tocopherol concentrations by d- α-tocopherol injection even though the other tissue α-tocopherol concentrations increased. Lauridsen et al [13] reported that during the first week of life the piglets from sows orally administered with deuterated vitamin E (d3-RRR-α-tocopheryl and d6-all-rac-α-tocopheryl acetates) from d 108 of pregnancy to d 7 of lactation acquired the labeled α-tocopherol from sow milk in order of liver>lung>heart>kidney> brain on an organ basis. In suckling rats from dams fed 0.1 or 1 g/kg diet of all-rac-α-tocopheryl acetate, brain α-tocopherol concentrations were not different even though liver and plasma α-tocopherol concentrations increased in the rats suckling from dams fed diets with greater vitamin E concentrations [14]. The current study results agreed with these previous findings indicating that injected vitamin E was distributed to various tissues that are critically important for pigs but brain may have a mechanism that controls α-tocopherol concentrations [13] and vitamin E accumulation into brain may take a longer time than in the other tissues [15].

Additionally, the α-tocopherol concentrations decreased from d 7 to 14 post-injection in liver, muscle, heart, and lung but not in brain. These changes of α-tocopherol concentrations in the current study had a similar pattern with those found by Lauridsen et al [13], in which deuterated α-tocopherol concentrations in pig brain were greater at d 21 of age than at d 7 whereas all other tissues (liver, muscle, heart, and lung) showed a reduction from d 7 of age to d 21.

In conclusion, fat-soluble vitamin (A, D_3_, and E) injection increased plasma status of α-tocopherol, retinol, retinyl palmitate, and 25-OHD_3_ concentrations and also increased vitamin A level in liver and vitamin E level in muscle, heart and liver. As plasma levels decreased from the peak after injection, the α-tocopherol found in plasma after administration was distributed to all other critically important tissues of pigs except for brain but retinyl palmitate only to the liver, which is a primary organ of vitamin A in the body.

## Figures and Tables

**Table 1:** Diet formulation and calculated chemical composition

Items	
Ingredients (%)	
Corn	43.71
Soybean meal (48% CP)	31.60
Fish meal	3.00
Blood meal	2.00
Whey, dried	15.00
Choice white grease	2.05
L-lysine·HCl	0.05
DL-methionine	0.18
L-threonine	0.13
Dicalcium phosphate	0.74
Limestone	0.82
Salt	0.50
Trace mineral premix1)	0.15
Vitamin premix without vitamin A2)	0.03
Choline chloride	0.03
Calculated chemical composition	
Metabolizable energy (kcal/kg)	3,402
Crude protein (%)	24.42
SID lysine (%)	1.35
SID methionine+cysteine (%)	0.84
SID Threonine (%)	0.95
Total Ca (%)	0.81
STTD P (%)	0.43

CP, crude protein; SID, standardized ileal digestible; STTD, standardized total tract digestible.

1) The trace mineral premix supplied the following per kilogram of diet: 50.0 mg of Mn as manganese sulfate, 100 mg of Fe as ferrous sulfate, 125 mg of Zn as zinc sulfate, 20.0 mg of Cu as copper sulfate, 0.35 mg of I as calcium iodate, and 0.30 mg of Se as sodium selenite.

2) The vitamin premix supplied the following per kilogram of diet: 1,500 IU of vitamin D_3_, 60 IU of vitamin E, 2 mg of vitamin K, 0.03 mg of vitamin B_12_, 7 mg of riboflavin, 25 mg of pantothenic acid, 20 mg of niacin, 1 mg of folic acid, 2.5 mg of vitamin B_6_, 2 mg of thiamin, and 0.15 mg of biotin. No vitamin A source was provided by the vitamin premix.

**Table 2:** Growth performance of pigs administered with fat-soluble vitamins

Items	Treatment1)		SEM	p-value

	Control	VITA		
Body weight (kg)				
d 0 post-injection2)	7.20	7.10	0.51	0.737
d 7 post-injection2)	9.20	8.77	0.60	0.381
d 14 post-injection3)	13.28	12.75	0.76	0.279
Average daily gain (kg/d)				
d 0–7 post-injection2)	0.287	0.239	0.03	0.134
d 7–14 post-injection3)	0.546	0.470	0.04	0.279

SEM, standard error of the mean.

1) Control, no fat-soluble vitamin administration; VITA, 300,000 IU of retinyl palmitate, 900 IU of d-α-tocopherol and 30,000 IU of vitamin D_3_ administration.

2) n = 8 per treatment.

3) n = 5 per treatment after 3 pigs per treatment were slaughtered for tissue collection and d 7 body weight of remaining pigs was used as a covariate.

**Table 3:** Retinol, retinyl palmitate, α-tocopherol, and 25OHD_3_ concentrations in plasma of pigs administered with fat-soluble vitamins1)

Items	Treatment2)		SEM	p-value

	Control	VITA		
Retinol3) (μg/mL)				
d 0 post-injection4)	0.11	0.08	0.01	0.005
d 3 post-injection4)	0.09	0.18	0.01	<0.001
d 7 post-injection4)	0.11	0.13	0.01	0.035
d 14 post-injection5)	0.14	0.17	0.01	0.002
Retinyl palmitate (μg/mL)				
d 0 post-injection4)	ND	ND	-	-
d 3 post-injection4)	ND	115.51	13.69	-
d 7 post-injection4)	ND	4.97	1.09	-
d 14 post-injection5)	ND	ND	-	-
α-tocopherol (μg/mL)				
d 0 post-injection4)	1.69	1.64	0.12	0.768
d 3 post-injection4)	0.99	17.14	1.19	<0.001
d 7 post-injection4)	0.80	2.35	0.14	<0.001
d 14 post-injection5)	0.66	0.99	0.28	0.408
25-OHD_3_ (ng/mL)				
d 0 post-injection4)	4.33	4.01	0.43	0.579
d 3 post-injection4)	3.44	71.00	6.98	<0.001
d 7 post-injection4)	4.79	36.35	3.26	<0.001
d 14 post-injection5)	7.24	15.50	1.22	<0.001

25OHD_3_, 25-hydroxycholecalciferol; SEM, standard error of the mean; ND, not detected.

1) Repeated measures was used for data analysis. The treatment, day effects, and day×treatment interaction were observed for retinol, 25OHD_3_, and α-tocopherol concentrations (p<0.01).

2) Control, no fat-soluble vitamin administration; VITA, 300,000 IU of retinyl palmitate, 900 IU of d-α-tocopherol and 30,000 IU of vitamin D_3_ administration.

3) The d 0 (initial) value was used as a covariate because of a significant difference between treatments.

4) n = 8 per treatment.

5) n = 5 per treatment after 3 pigs per treatment were slaughtered for tissue collection.

**Table 4:** Retinol and retinyl palmitate concentrations in liver of pigs administered with fat-soluble vitamins

Items	Treatment1)		SEM	p-value

	Control	VITA		
Retinol (μg/g)				
d 0 post-injection2)	0.85	-	0.16	-
d 7 post-injection3)	0.63	19.10	1.87	0.020
d 14 post-injection3)	0.33	1.37	0.07	0.004
Retinyl palmitate (μg/g)				
d 0 post-injection2)	54.10	-	7.10	-
d 7 post-injection3)	43.33	199.13	12.52	0.013
d 14 post-injection3)	20.30	140.87	4.15	0.002
Retinol+retinyl palmitate4) (RE μg/g)				
d 0 post-injection2)	30.41	-	3.98	
d 7 post-injection3)	24.31	127.92	6.74	0.008
d 14 post-injection3)	11.43	78.34	2.45	0.002

SEM, standard error of the mean; RE, retinol equivalents.

1) Control, no fat-soluble vitamin administration; VITA, 300,000 IU of retinyl palmitate, 900 IU of d-α-tocopherol and 30,000 IU of vitamin D_3_ administration.

2) n = 4 for the initial value.

3) n = 3 per treatment.

4) The values were calculated by summing retinol and retinyl palmitate concentrations after conversion to retinol equivalents.

**Table 5:** Alpha-tocopherol concentrations in liver, muscle, heart, lung and brain of pigs administered with fat-soluble vitamins

Items	Treatment1)		SEM	p-value

	Control	VITA		
Liver (μg/g)				
d 0 post-injection2)	3.98	-	0.20	-
d 7 post-injection3)	4.30	11.17	1.43	0.074
d 14 post-injection3)	1.90	3.73	1.06	0.345
Muscle (μg/g)				
d 0 post-injection2)	3.73	-	0.44	-
d 7 post-injection3)	3.07	8.30	0.77	0.040
d 14 post-injection3)	2.80	3.90	0.37	0.172
Heart (μg/g)				
d 0 post-injection2)	2.88	-	0.49	-
d 7 post-injection3)	4.60	24.80	4.54	0.088
d 14 post-injection3)	2.17	4.57	0.55	0.092
Lung (μg/g)				
d 0 post-injection2)	10.08	-	0.79	-
d 7 post-injection3)	14.40	18.80	6.52	0.680
d 14 post-injection3)	9.60	10.93	1.07	0.472
Brain (μg/g)				
d 0 post-injection2)	5.93	-	0.38	-
d 7 post-injection3)	8.20	7.57	1.54	0.326
d 14 post-injection3),4)	7.37	8.33	0.36	0.281

SEM, standard error of the mean.

1) Control, no fat-soluble vitamin administration; VITA, 300,000 IU of retinyl palmitate, 900 IU of d-α-tocopherol and 30,000 IU of vitamin D_3_ administration.

2) n = 4 for the initial value.

3) n = 3 per treatment.

4) One sample was removed from the Control treatment as an outlier.
